# Urinary tract infections (UTIs) or genital tract infections (GTIs)? It's the diagnostics that count

**DOI:** 10.3205/dgkh000320

**Published:** 2019-02-18

**Authors:** Payam Behzadi, Elham Behzadi, Edyta Agnieszka Pawlak-Adamska

**Affiliations:** 1 Department of Microbiology, College of Basic Sciences, Shahr-e-Qods Branch, Islamic Azad University, Tehran, Iran; 2 Department of Experimental Therapy, Hirszfeld Institute of Immunology and Experimental Therapy, Wroclaw, Poland

**Keywords:** sexual intercourse, urinary tract infections, genital tract infections, toll-like receptors

## Abstract

Urinary tract infections (UTIs) and genital tract infections (GTIs) are both very common infectious diseases. Thus, accuracy and rapidity in recognition and treatment of sexually transmitted urogenital tract infections (ST-UGTIs) is a major concern in global public health systems. The application of reliable, accurate diagnostic tools is the key to definite detection, identification and treatment. This literature review focused on different characteristics of UGTIs in patients and the importance of diagnostic methodologies.

The articles published and indexed from 1980 through October 2018 in the databases of PubMed and MEDLINE, as well as the Google Scholar web search engine, were collected and studied. MeSH keywords of “Sexual intercourse”, “Urinary Tract Infections”, “Genital Tract Infections” and “Toll-Like Receptors” were used for searching articles. Then, the proper articles (original and review articles) were subjected to a very rigorous selection process.

The clinical symptoms and signs or asymptomatic properties of UTIs and GTIs are similar and often overlap. In many cases, the lack of suitable diagnostic techniques leads to misdiagnosed/undignosed GTIs and overdiagnosed UTIs. The outcome of poor diagnostics is failure of definite identification and treatment.

The application of advanced techniques comprising PCR, microarray and next-generation sequencing promises to be more effective, together with the use of the microbial pattern of the individual’s UGT to provide reliable detection, identification and definite treatment. This will be an option in the near future.

## Zusammenfassung

Infektionen des Harntrakts (HWI) und des Genitaltrakts (GTI) sind beides infektiöse Erkrankungen. Deshalb ist die Genauigkeit und Schnelligkeit der Erkennung und Behandlung sexuell übertragener Infektionen des Urogenitaltrakts (SÜ-UGT) entscheidend. Die Verwendung geeigneter Diagnosetools ist der Schlüssel zur eindeutigen Erkennung, Identifizierung und Behandlung. Die Literaturrecherche konzentrierte sich auf verschiedene Merkmale von SÜ-UGTs und die Bedeutung diagnostischer Methoden.

Veröffentlichungen (Originalbeiträge und Reviews) wurden im Zeitraum von 1980 bis Oktober 2018 in den Datenbanken PubMed, MEDLINE und Google Scholar anhand der Schlüsselwörter „Sexual intercourse“, „Urinary Tract Infections“, „Genital Tract Infections“ und „Toll-Like Receptors“ gesammelt, sorgfältig ausgewählt und ausgewertet.

HWI und GTI überlappen sich häufig entweder auf Grund der ähnlichen klinischen Symptomatik oder wegen asymptomatischen Verlaufs. In vielen Fällen führt das Fehlen geeigneter Diagnosemethoden zu falsch diagnostizierten oder nicht erkannten GTI und überdiagnostizierten HWI. Das Ergebnis einer unzureichenden Diagnostik führt zum Versagen der eindeutigen Identifizierung und Behandlung.

Die Anwendung moderner Techniken wie PCR, Microarray und Next-Generation-Sequenzierung wird in Verbindung mit der Identifizierung der mikrobiellen individuellen Besiedlung des UGT eine qualitativ hochwertige Erkennung und Behandlung ermöglichen.

## Introduction

Urinary tract infections (UTIs) are the second most common infectious diseases worldwide. UTIs involve a wide range of clinical manifestations, including acute, chronic, uncomplicated, complicated, asymptomatic, symptomatic and recurrent. The infections may affect the lower and/or the upper parts of urinary tract. There are different types of microbial pathogens, such as Gram-negative bacteria (e.g., uropathogenic *Escherichia coli* [UPEC]), Gram-positive bacteria (e.g., *Staphylococcus saprophyticus*) and yeasts (e.g., *Candida albicans*), which may cause UTIs in their hosts. Urogenital tract infections (UGTIs) include the prevalent infectious diseases of the genital tract, urinary tract and sexually transmitted urogenital tract infections (ST-UTIs), which have become a major concern in public health systems [1], [2], [3], [4], [5], [6], [7], [8], [9], [10], [11], [12].

UTIs such as cystitis (lower UTIs) and pyelonephritis (upper UTIs) in healthy hosts with no history of UTIs are recognized as uncomplicated UTIs. In contrast, complicated UTIs are normally seen in the elderly and catheterized patients. Although there is a wide range of predisposing factors which may lead to the occurence of UTIs, the uncomplicated cases are mostly known as ST-UTIs. UTIs and GTIs sometimes occur together, and the GTIs are often ignored or misdiagnosed. In other words, there are many patients who suffer from UGTIs, but the GTIs are reported as UTIs. Thus, the misdiagnosis/underdiagnosis of GTIs and overdiagnosis of UTIs is a serious error, leading to incorrect treatment and spread of infections. Furthermore, asymptomatic UGTIs can be considered another significant problem in public health systems [8], [13], [14], [15], [16], [17], [18], [19].

Based on the high frequency of UGTIs and the severe consequences of misdiagnosis, this literature review focused on different characteristics of UGTIs in patients and the importance of diagnostic methodologies.

## Methods

The articles published and indexed from 1980 through October 2018 in the databases of PubMed and MEDLINE as well as the Google Scholar web search engine were collected and studied. The MeSH keywords “sexual intercourse”, “Urinary Tract Infections”, “Genital Tract Infections” and “Toll-Like Receptors” were used for searching articles. Then, the proper articles (original and review articles) were selected very rigorously. 

## Results and discussion

### Sexual intercourse, UTIs, and GTIs

Adolescence results in physical and emotional maturity. This process may lead to special social and intimate relationships between young people and adults. Sexual intercourse experimentation is one of the most important needs among teenagers, young adults, and adults. Uncertain and uncontrolled sexual activities and sexual intercourse may lead to serious health problems and the occurrence of UGTIs. Venereal diseases (VD) and ST-UGTIs occur through anal, oral and vaginal sexual activities. Cystitis and rarely pyelonephritis caused by UPEC are known as frequent UTIs in sexually active women. As previous studies have shown [16], [18], [19], [20], [21], the number of misdiagnosed/undiagnosed GTIs and overdiagnosed UTIs among sexual active women is extremely high. According to reports from the Centers for Disease Control and Prevention (CDC) [18], about 19 million cases are added annually to the population of patients with STIs. Because of the damaging epidemiological consequences of STIs, performing early diagnosis and definite treatment are imperative. The major problem is that in UGTIs, there is no distinct border between GTIs and UTIs. According to the literature [16], [20], [21], in up to 50% of patients with UTIs, GTIs were also recognized (UGTIs). Moreover, there are several symptoms common to the early stages of both GTIs and UTIs. However, many UGTIs are clinically asymptomatic. Sometimes, important pathogenic bacteria, such as *Gardnerella vaginalis*, *Ureaplasma* spp. and *Mycoplasma hominis*, are actually members of the UT’s normal flora but act as pathogenic microorganisms in the etiology of UGTIs (in some cases, *Ureaplasma* spp. and* Mycoplasma hominis* are known as UGT commensal bacteria). Thus, possessing an up-to-date microbiota pattern from UGTs for differentiating pathogenic microorganisms from commensal strains helps us to estimate the real status of UGTIs among populations. There are different types of GTIs which have close similarities with UTIs. Table 1 (Tab. 1) describes these infections and their characteristics [1], [5], [12], [16], [17], [19], [22], [23], [24], [20], [21], [25], [26], [27], [28], [29], [30], [31], [32], [33], [34], [35], [36], [37], [38], [39], [40], [41], [42], [43], [44], [45].

### Risk factors for UGTIs

A wide range of predisposing factors contributes to multifactorial ST-UGTIs. The most commonly reported risk factors which predispose people to ST-UGTIs are: early sexual activity, frequency of coition, frequency of voiding before and after coition, sexual intercourse with different sex partners (separately or concurrently), sexual abuse, rape, sexual intercourse with addicted partners, sexual intercourse with sex workers, sexual intercourse with online dating friends, sexual intercourse with a new sex partner within less than 2 months, history of previous GTIs or UTIs, immune deficiencies, sexual intercourse with gay, lesbian or bisexual partners, low socioeconomic status, depression, anxiety, low educational status, poor personal hygiene, poor access to condoms and other contraceptive devices, use of spermicidal diaphragms (spermicidal agents also kill lactobacilli), poor access to qualified healthcare systems, high rate of family disruption, and mental disorders. In addition, the increase and spread of multidrug-resistant pathogens and expanded spectrum beta lactamase (ESBL-)producing bacteria have a considerable impact on UGTIs. However, some studies suggest an interesting methodology for overcoming the problem of ESBL-producing enterobacteriaceae by individualized therapy. In other words, the profiles of suscepti-bilities, severities and types of UGTIs and the characteristics of patients differ from one patient to the next. These properties open promising approaches to providing accurate and definite treatment for UGTIs [22], [23], [24], [46], [47], [48], [49]. 

### Important TLRs (toll-like receptors) in UGTIs

**Figure 1:**
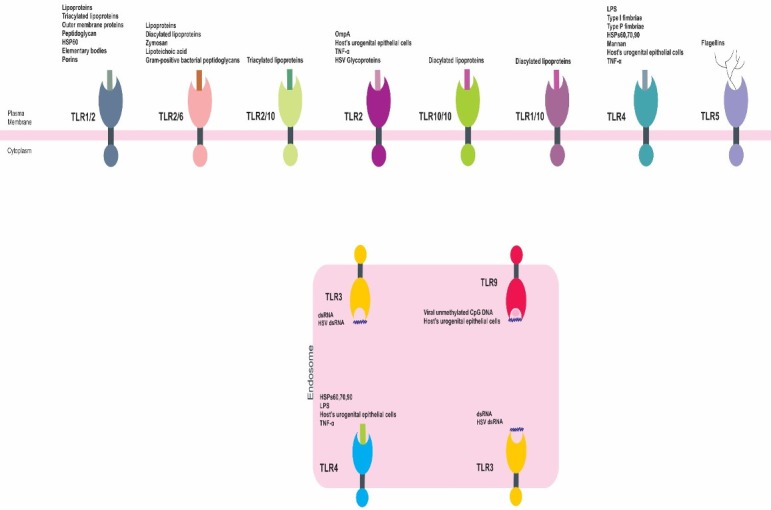
The situation, arrangement and relationship between pathogen-associated molecular patterns (PAMPs) and TLRs in UGTIs

Despite the presence of strong barriers made of urothelial cells in human UGT, uropathogenic microorganisms such as UPEC can breach these barriers. Upon the entrance of UPEC into UGT, the innate immune responses are activated by the expression of certain toll-like receptors (TLRs) within the urothelial cells of bladder and kidneys. TLR expression activates a cascade of different immune system components including chemokines, interferons, interleukins, antimicrobial peptides, and proinflammatory cytokines. The innate immune system responds upon the occurrence of by UPEC entrance, attachment and invasion. The bacterial attachment on the urothelial cells is achieved by the virulence factors of FimH adhesins lo-cated on the top of type I fimbriae. The attachment of UPEC cells on the urothelium cells normally leads to expression of TLRs, secretion of interleukin (IL-)6 and the function of programmed cell death (apoptosis; genome fragmentation), where apoptosis may prevent the process of bacterial invasion into the UT epithelial cells and act as a mechanism of immediate bacterial elimination. The UPEC cells are normally recognized by TLRs 4 and 5. According to several surveys [42], [50], [51], the molecules of TLR4 are expressed by bladder and kidney urothelial cells, while TLR5 molecules are expressed via bladder urothelial cells. The attachment of bacterial lipopolysaccharides (LPS) or P fimbriae to CD14 and glygoprotein molecules (situated on uroplakins/cell membrane of bladder and kidney urothelial cells), respectively, results in TLR4 expression, while the bacterial flagella units (flagellins) activate the expression of TLR5 molecules in bladder urothelial cells. Activation of TLR4 may trigger IL-8 and IL-6 secretion. IL-8 and IL-6 cytokines contribute to neutrophil recruitment and secretion of mucosal antibody of IgA *in situ*, respectively. In addition to UPEC as an important microbial causative agent of UGTIs, fungi such as *C. albicans* are important pathogens that cause UGTIs. Interestingly, *C. albicans* is able to stimulate the innate immune responses via different types of TLRs, including TLR2/6 (zymosan) and TLR4 (neutrophil activation and secretion of mucosal antibody of IgA). The relationship between microbial causative agents of UGTIs and TLRs are shown in Figure 1 (Fig. 1) and Table 2 (Tab. 2) [2], [3], [42], [50], [51], [52], [53], [54], [55], [56], [57], [58], [59], [60], [61], [62], [63], [64], [65], [66], [67], [68].

### Epidemiology of UGTIs

Patients with UTIs are referred to 7,000,000 medical practices and 1,000,000 emergency departments worldwide. In addition to patients with UTIs, the patients with GTIs are also referred to these medical centers. In accordance with CDC reports, about 20,000,000 patients with STIs are recognized as new cases annually. There are several clinical manifestations common to both GTIs and UTIs (in particular lower UTIs). At the same time, there are several types of UGTIs which are asymptomatic. In addition to these important problems, knowledge about microbial load and population is critical for the detection and identification of pathogenic agents of UGTIs. Healthy people have UGT microbiota with their own UGT microbial patterns. In patients with UGTIs, the microbiotal ecosystem becomes unbalanced, leading to the occurrence of UGTIs. In the near future, knowing the microbial patterns of healthy UGT and UGTIs will be an invaluable and effective biodiagnostic tool. That is why a large proportion of patients with GTIs undergo wrong treatments and are undiagnosed or misdiagnosed. Today, we know that the reports regarding UTIs and GTIs are neither accurate nor correct. It seems that the reported percentage of patients with UTIs (about 50%) or GTIs (up to 50%) is biased. Overdiagnosis and underdiagnosis have occurred relating to the rates of UTIs and GTIs, respectively. The use of advanced molecular and pan-genomic diagnostic methods, such as polymerase chain reaction (PCR) and DNA microarray, is more appropriate than traditional microbiological diagnostic tests. In addition, the application of sequencing technologies, e.g., 16s rRNA sequencing, is recommended for viable but non-culturable microorganisms. Normally, the PCR technique is applied to detect and identify a limited number of genes and samples, while the DNA microarray technique is applied for large numbers of genes and samples. Using 16s rRNA sequencing technologies has offered us an opportunity to detect and identify those microorganisms which do not grow on lab culture media. On the other hand, some recommendations for urogenital pathogen cutoffs of colony forming units (cfu/ml) may lead to incorrect diagnosis. The guideline of the European Association of Urology (EAU) (cited in [30]) suggests a cutoff of ≥10^3^ to ≥10^5^ cfu/ml for women and ≥10^4^ cfu/ml for men as the standard critical number of uropathogenic microorganisms in midstream urine samples regarding bacteriuria. These cutoffs are completely dependent on the UGT microbial patterns. Thus, by employing state-of-the-art microbiological diagnostic methods, it will be possible to obtain a much more accurate interpretation of the results. Thus, the gold standard methodologies of microbiological techniques should be complemented by advanced molecular, pan-genomic and sequencing technologies. As mentioned above, PCR assays are cost-effective, fast, sensitive and specific for a limited number of genes and samples, but DNA microarray is preferable to reach an accurate, inexpensive, fast, flexible, reliable, sensitive and specific diagnosis given a large number of genes and specimens. 16s rRNA sequencing technology can provide a complete microbial pattern of the patient’s UGT microbiota. Thus, the patient’s anamnesis, clinical manifestations, diagnostic characteristics and application of appropriate diagnostic methods enable reliable detection and identification necessary for definite, appropriate treatment [1], [2], [9], [10], [12], [16], [17], [24], [30], [44], [45], [69], [70], [71].

### UGTIs categorizations

As Table 1 (Tab. 1) depicts, there is a wide range of microorganisms which cause different types of UGTIs. Despite considerably overlaps in UTIs and GTIs, an appropriate screening methodology for accurate clinical diagnosis and definite qualified treatment would facilitate differentiating UTIs from GTIs. As mentioned above, patient’s anamnesis, physical examinations, clinical syndromes, diagnostic tools, and regional lab information regarding local infections and the related pathogens are significant items for the detection and identification of UGTIs. Clinical characteristics, manifestations, signs and symptoms such as pyuria, dysuria, frequent urination, urgent urination, hematuria, pain in the flank and suprapubic region, fever and chills are common in both GTIs and UTIs. Moreover, penile discharge in men and vaginal discharge in women may occur in UTIs, but not always. In contrast to UTIs, GTIs are often recognized by genital tract discharges. Therefore, the type of clinical diagnostic tools determines the quality of UGTI recognition [1], [4], [10], [12], [16], [17], [19], [24], [30], [31], [44], [45], [70].

### UGT microbiota

Today, a wide spectrum of possibilities and insights are available regarding UGT microbiota. The use of different molecular, nucleic-acid-based, pan-genomic and next-generation technologies enables improves the quality of information about human UGT microbiota. Indeed by the beginning of the human microbiome (microbial genomic treasure) project, the knowledge has greatly increased about an individual’s UGT microbiotic profile. At present, we know that the UGT has its own microbiota and it differs not only between men and women but also from person to person. There are many viable but non-culturable microorganisms which can be identified throughout advanced molecular tools, sequencing techniques and microarray technologies. The useful UGT microbiota guarantee the health of the UGT, and any changes in the microbial pattern of UGT microbiota leads to imbalance and the occurrence of UGTIs. In the near future, the microbial pattern of UGTs will become invaluable biomarkers. It seems that human UGT microbiota also differ in different geographical regions; moreover, the effect of an individual’s behaviors on his/her UGT microbiota is detectable. For example, the microbial patterns of UGTs in individuals who have frequent sexual intercourse with different partners are more variable than in people with normal sexual activities. Each sex partner shares his/her UGT microbiota with the other. As reported previously, the use of sex toys by women may result in a decrease of the vaginal population of *Lactobacillus* spp. and increase in *G. vaginalis*. In toto, the reduction of the *Lactobacillus* population may provide a favorable environment for pathogenic microorganisms. This means that *Lactobacillus* spp. are sentinel biobarriers which stabilize the UGT environment and keep it healthy. *Lactobacillus* spp. prevent the occurrence of ST-UGTIs in women. Previous reports show that *Lactobacillus crispatus* in a healthy vaginal environment traps viral particles of HIV in cervicovaginal mucus, and eliminates pathogenic bacteria of *N. gonorrhoeae* and UPEC. The presence of *lactobacilli* prevents recurrent UGTIs in susceptible women. Furthermore, bactericidal effects of *lactobacilli* on *C. trachomatis* is reported. *C.* *trachomatis* is the major bacterial causative agent of STIs around the world. In some cases, such as the occurrence of trichomoniasis, the presence of *T. vaginalis* is correlated with the presence of *Mycoplasma* spp., e.g., *M. hominis*, and the reduction of *L. crispatus* populations ([1], [3], [9], [44], [45], [70], [71]).

Despite the variation of microbial patterns of UGTs at different ages in both men and women, there are great similarities in their microbial patterns of UGTs within a certain age range (similar microbial patterns in age ranges of 24–50 years [70], [72], and 39–86 years (in men) [70], [73], 22–51 years old [70], [72], and 26–90 years old (in women) [70], [73]). Table 3 (Tab. 3) depicts the total populations of UGT microbiota in both men and women [44], [45], [70].

For patients with asymptomatic UGTIs, several studies have found that determining their microbial patterns can offer a solution. For example, in the presence of asymptomatic UGTIs caused by *C. trachomatis*, the population of useful bacteria such as *lactobicilli* decreases in the cervicovagina, while the population of anaerobic bacteria such as *G. vaginalis* increases [44], [45], [70].

This shows that the application of accurate, rapid, sensitive and specific diagnostic tools and technologies definitely has a direct effect on our ability to detect and identify UGTIs. Therefore, knowledge about the quality and the influence of relevant diagnostic techniques is imperative. Some common and important diagnostic methods are discussed as below. 

### Laboratory diagnostics

In parallel with clinical signs and symptoms, physical tests and patient’s history, the use of appropriate laboratory diagnostic tools is necessary. It is clear that a clean midstream urine sample, penile discharges and vaginal discharges, bleeding and lesions are required to provide an accurate and definite diagnosis. Dipsticks, microscopic observations, microbiological cultivation, test strips, biomarkers, flow cytometry, mass spectrometry, infrared spectroscopy, isothermal microcalorimetry, biosensors, nucleic-acid-based techniques (including PCR), microarray, and sequencing technologies are some of the modern diagnostic techniques and microbial biomarkers. Of course, the use of appropriate diagnostic tools enables us to detect UGTIs with minimal biases [1], [4], [6], [7], [12], [16], [30], [31], [45], [69], [70], [71]. In the following text, these techniques are described in brief.

Dipstick tests are common diagnostic methods for the detection and identification of uropathogenic bacteria, including UPEC and other enteric bacteria. These assays are known as screening tests for UGTIs to recognize inflammations in the UGT and bacteriuria by detecting leukocyte estrase enzyme (produced by leukocytes) and nitrite (produced by enteric bacteria) in the urine samples. The results of these techniques can help us to confirm or exclude the presence of UGTIs. The sensitivity and specificity of dipstick assays are reported as 77% and 70%, respectively. It is clear that negative results from dipstick tests should be processed for further assessment, including microscopic observation, Gram staining and microbiological cultivation. The negative results can be interpreted as an alert for asymptomatic bacteriuria or GTIs [1], [12], [16], [31], [69].

Microscopic observation is usually performed together with Gram staining procedures. These methods are other elements of screening tests for recognition of UGTIs. It is important to check clinical samples as early as possible, because the number of leukocytes decreases at lab temperature over time (an approximately 40% decrease within 2 hours). Pyuria (identified as 5 leukocytes in a high-power microscopic field) is considered for UGTIs. The morning urine samples are preferred for a narrower diagnosis. The microscopic examination of the sample must be done in parallel with other complementary assays for detection and identification of UGTIs. Pyuria is an important alert for GTIs, if the other results have excluded UTIs [1], [4], [16], [18], [69].

Microbiological cultivation is a gold standard for bacteriuria (within 24 hours). The use of different important culture media, including blood agar and MacConkey agar, is useful. Cultivation is an important tool for detecting and identifying different types of culturable pathogens. The results may show multimicrobial infectious diseases of UGTIs. However, this technique has its limitation regarding sample contamination, viable but non-culturable microorganisms, patients’ skills in sampling, anatomical problems and disorders. Positive results of non-invasive and invasive specimens are recognized as 10^4^–10^5^ and 10^2^ cfuU/ml, respectively [1], [4], [12], [16], [30], [31], [69].

The test strip is a kind of ELISA based on paper containing antibodies against microbial causative agents of UGTIs. The test strip covers the weak points of dipstick specificity. For detecting and identifying microbial causative agents of UGTIs, this assay is easy to do, effective, and inexpensive [69]. 

There is a wide range of biomarkers (immunomarkers) which can be used for recognition of UGTIs. For instance, C-reactive protein (CRP), interleukins, cytokines, chemokines, TLRs, antibodies and others are appropriate for the detection and identification of UGTIs [16], [47], [69].

Flow cytometry is a technique based on laser illumination and tracing the scattered beam. The results are obtained by measuring scattered light beams. In this method, the urine is directly checked for pathogenic microorganisms, red blood cells (RBCs) and white blood cells (WBC). The sensitivity and specificity of flow cytometry is around 90% and 65%, respectively. This technique can be used as a single screening tool for recognition of UGTIs, but the cutoff of cfu/ml is individually determined for different microbial species and patient categories [1], [69].

Mass spectrophotometry is another routine technique for directly testing urine samples. Matrix-assisted laser desorption ionization time-of-flight mass spectrophotometry (MALDI TOF MS) can be used to identify Gram-negative and Gram-positive bacteria. The sensitivity and specificity of this technique are high for Gram-negative bacteria [69]. Thus, this technique is useful for UGTIs caused by Gram-negative bacteria.

The electromagnetic excitation and light scattering technique of infrared spectroscopy works on cultured samples of bacterial cells. The sensitivity and specificity of infrared spectroscopy for Gram-positive bacteria is 100%. This methodology is useful for determining multi-drug resistant (MDR) bacteria [69] and detection of UGTIs.

The isothermal microcalorimetry technique is a quick-time method for detection and identification of microbial agents of UGTIs. There is a specific cfu/ml limit for each microbial agent. Despite some advantages of isothermal microcalorimetry assay, this tool is dependent on cultivation [69].

The assays based on biosensors are effective, rapid, and have high specificity and sensitivity. This method is useful for directly testing urine samples. Biosensor-based technologies have limits for microbial cfu/ml; hence, it varies for different microbial species. The importance of this technique lies in its ability to differentiate between live and dead microbial cells. Biosensors are also able to detect MDR microbial species. The biosensor-based techniques must be set up for each microbial species [69]. Therefore, it seems that this technology may be useful for reliable detection and identification of UGTIs.

Today, there are wide ranges of nucleic-acid-based techniques including PCR, microarray and next-generation sequencing technologies. Given culturable microorganisms with a limited number of clinical samples, the molecular tool PCR is recommended. But in the case of large numbers of genes and samples, the use of an advanced pan-genomic technology such as microarray is unavoidable. Simultaneously, there are vast populations of microorganisms which are viable but non-culturable; these groups of microorganisms are recognized only through sequencing techniques, e.g., the 16s rRNA sequencing method. To have a real and accurate microbial pattern of related microorganisms including microbiota and pathogens, the employment of different technologies are recommended. In toto, the determination of microbial patterns to detect and identify microbial causative agents of UGTIs will be unavoidable in the near future. Thus, microbial biomarkers are our new options to provid high quality detection and identification of UGTIs [2], [3], [9], [10], [16], [17], [45], [68], [69], [70], [71].

## Conclusion

UGTIs are one of the most common infectious diseases, which affects millions of people around the world. Despite the global concerns about UGTIs both in terms of costs and public health systems, there are many problems associated with the UGTI detection, identification and definite treatment. Although familiarity with the clinical manifestations of UGTIs, the related predisposing factors, patient’s private history, the application of gold standard and routine diagnostic methods is needed for successful detection, identification and definite treatment, there is still a dramatic lack of accuracy in the detection, identification and definite treatment of UGTIs. In recent years, advanced diagnostic techniques have become accessible but the level of diagnostic biases is high. Depending on the obtained microbial patterns for an individual’s UGT, the application of advanced techniques comprising PCR, microarray and next-generation sequencing will be more effective together with the microbial pattern of an individual’s UGT in providing quality detection, identification and definite treatment. 

## Notes

### Conflicts of interest

The authors declare no conflicts of interest.

## Figures and Tables

**Table 1:**
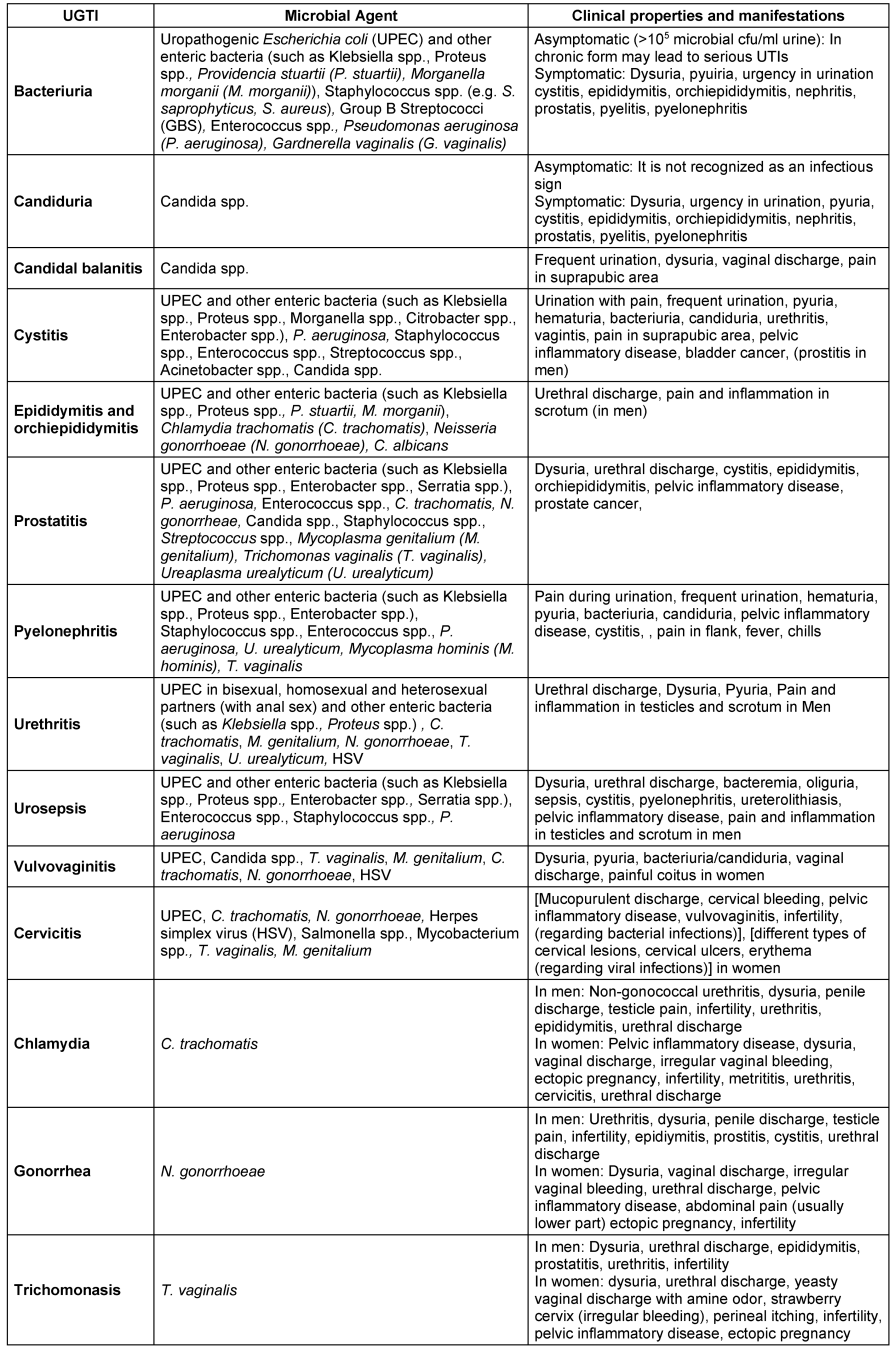
Urogenital tract infections (UGTIs), microbial causative agents and related clinical properties and manifestations

**Table 2:**
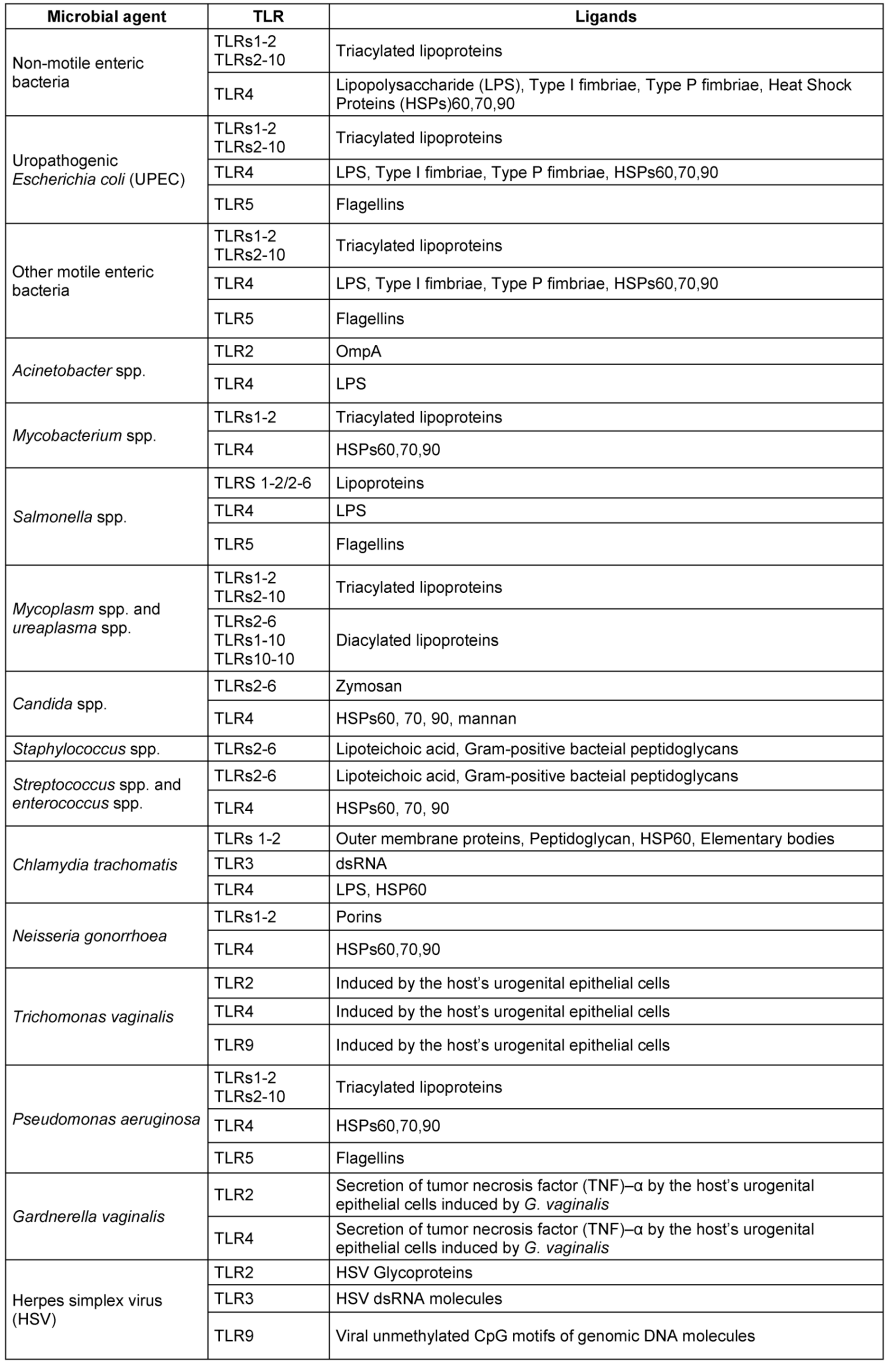
The relationship between toll-like receptor (TLR) induction and microbial causative agents of UGTIs in humans

**Table 3:**
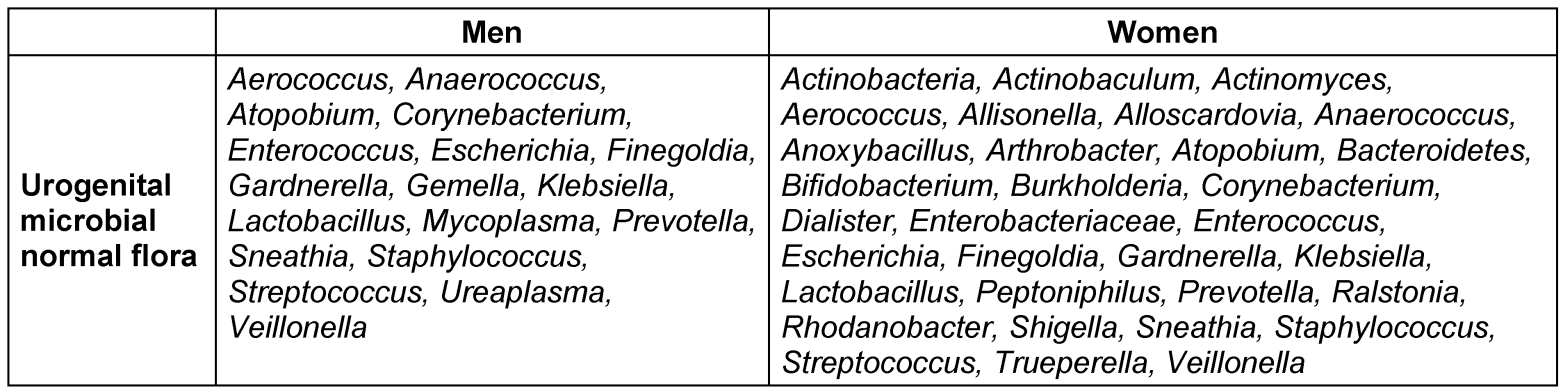
The population of UGT microbiota in healthy men and women

## References

[1] Oyaert M, Van Meensel B, Cartuyvels R, Frans J, Laffut W, Vandecandelaere P, De Beenhouwer H, BILULU Study Group (2018). Laboratory diagnosis of urinary tract infections: Towards a BILULU consensus guideline. J Microbiol Methods.

[2] Behzadi P, Jarzembowski T (2018). Uropathogenic Escherichia coli and Fimbrial Adhesins Virulome. Urinary Tract Infection: The Result of the Strength of the Pathogen, or the Weakness of the Host.

[3] Behzadi P, Behzadi E, Rojas I, Ortuño F (2017). Uropathogenic Escherichia coli: An Ideal Resource for DNA Microarray Probe Designing. Bioinformatics and Biomedical Engineering - IWBBIO 2017- Lecture Notes in Computer Science (vol 10209).

[4] Behzadi P, Behzadi E (2008). The Microbial Agents of Urinary Tract Infections at Central Laboratory of Dr. Shariati Hospital, Tehran, Iran. Turk Klin Tip Bilim.

[5] Behzadi P, Behzadi E, Ranjbar R (2015). Urinary tract infections and Candida albicans. Cent European J Urol.

[6] Behzadi P, Behzadi E, Yazdanbod H, Aghapour R, Akbari Cheshmeh M, Salehian Omran D (2010). A survey on urinary tract infections associated with the three most common uropathogenic bacteria. Maedica (Buchar).

[7] Behzadi P, Behzadi E, Yazdanbod H, Aghapour R, Akbari Cheshmeh M, Salehian Omran D (2010). Urinary Tract Infections Associated with Candida albicans. Maedica (Buchar).

[8] Kline KA, Lewis AL (2016). Gram-Positive Uropathogens, Polymicrobial Urinary Tract Infection, and the Emerging Microbiota of the Urinary Tract. Microbiol Spectr.

[9] Jahandeh N, Ranjbar R, Behzadi P, Behzadi E (2015). Uropathogenic Escherichia coli virulence genes: invaluable approaches for designing DNA microarray probes. Cent European J Urol.

[10] Behzadi P, Najafi A, Behzadi E, Ranjbar R (2016). Microarray long oligo probe designing for Escherichia coli: an in-silico DNA marker extraction. Cent European J Urol.

[11] Ranjbar R, Tabatabaee A, Behzadi P, Kheiri R (2017). Enterobacterial Repetitive Intergenic Consensus Polymerase Chain Reaction (ERIC-PCR) Genotyping of Escherichia coli Strains Isolated from Different Animal Stool Specimens. Iran J Pathol.

[12] Choe HS, Lee SJ, Yang SS, Hamasuna R, Yamamoto S, Cho YH, Matsumoto T, Committee for Development of the UAA-AAUS Guidelines for UTI and STI (2018). Summary of the UAA-AAUS guidelines for urinary tract infections. Int J Urol.

[13] Hooton TM (2012). Clinical practice. Uncomplicated urinary tract infection. N Engl J Med.

[14] Neal DE (2008). Complicated urinary tract infections. Urol Clin North Am.

[15] Flores-Mireles AL, Walker JN, Caparon M, Hultgren SJ (2015). Urinary tract infections: epidemiology, mechanisms of infection and treatment options. Nat Rev Microbiol.

[16] Tomas ME, Getman D, Donskey CJ, Hecker MT (2015). Overdiagnosis of Urinary Tract Infection and Underdiagnosis of Sexually Transmitted Infection in Adult Women Presenting to an Emergency Department. J Clin Microbiol.

[17] Sarier M, Sepin Ozen N, Guler H, Duman I, Yüksel Y, Tekin S, Yavuz AH, Yucetin L, Erdogan Yilmaz M (2018). Prevalence of Sexually Transmitted Diseases in Asymptomatic Renal Transplant Recipients. Exp Clin Transplant.

[18] Shipman SB, Risinger CR, Evans CM, Gilbertson CD, Hogan DE (2018). High Prevalence of Sterile Pyuria in the Setting of Sexually Transmitted Infection in Women Presenting to an Emergency Department. West J Emerg Med.

[19] Antinori S, Pezzani MD, Tonolini M (2018). Uncomplicated and complicated urinary tract infections in adults: The infectious diease's specialist perspective. Imaging and Intervention in Urinary Tract Infections and Urosepsis.

[20] Berg E, Benson DM, Haraszkiewicz P, Grieb J, McDonald J (1996). High prevalence of sexually transmitted diseases in women with urinary infections. Acad Emerg Med.

[21] Shapiro T, Dalton M, Hammock J, Lavery R, Matjucha J, Salo DF (2005). The prevalence of urinary tract infections and sexually transmitted disease in women with symptoms of a simple urinary tract infection stratified by low colony count criteria. Acad Emerg Med.

[22] Vanmullekom AM (2016). Sexually Transmitted Infections. Wiley Blackwell Encyclopedia of Family Studies.

[23] Holt M (2016). Sexually Transmitted Infections. Wiley Blackwell Encyclopedia of Gender and Sexuality Studies.

[24] Mermelstein S, Plax K (2016). Sexually transmitted infections. Curr Treat Options Peds.

[25] Dan M, Gottesman T, Schwartz O, Tsivian A, Gophna U, Rokney A (2012). Sexually transmitted Escherichia coli urethritis and orchiepididymitis. Sex Transm Dis.

[26] Ragnarsdóttir B, Svanborg C (2012). Susceptibility to acute pyelonephritis or asymptomatic bacteriuria: host-pathogen interaction in urinary tract infections. Pediatr Nephrol.

[27] Nicolle LE, Bradley S, Colgan R, Rice JC, Schaeffer A, Hooton TM, Infectious Diseases Society of America, American Society of Nephrology, American Geriatric Society (2005). Infectious Diseases Society of America guidelines for the diagnosis and treatment of asymptomatic bacteriuria in adults. Clin Infect Dis.

[28] Coker TJ, Dierfeldt DM (2016). Acute Bacterial Prostatitis: Diagnosis and Management. Am Fam Physician.

[29] Ollendorff AT (2017). Cervicitis. Medscape.

[30] Smelov V, Naber K, Johansen TEB (2016). Improved classification of urinary tract infection: Future considerations. Europ Urol Suppl.

[31] Matulay JT, Mlynarczyk CM, Cooper KL (2016). Urinary tract infections in women: Pathogenesis, diagnosis, and management. Curr Bladd Dysfunct Rep.

[32] Qureshi S (2018). Chlamydial genitourinary infections. Medscape.

[33] Haldar S, Dru C, Bhowmick NA (2014). Mechanisms of hemorrhagic cystitis. Am J Clin Exp Urol.

[34] Hooton TM, Gupta K (2017). Acute uncomplicated cystitis and pyelonephritis in women. UpToDate.

[35] Wagenlehner FM, Lichtenstern C, Rolfes C, Mayer K, Uhle F, Weidner W, Weigand MA (2013). Diagnosis and management for urosepsis. Int J Urol.

[36] Wagenlehner FME, Pilatz A, Weidner W, Naber KG (2015). Urosepsis: Overview of the Diagnostic and Treatment Challenges. Microbiol Spectr.

[37] Behzadi P, Behzadi E (2012). Evaluation of UVB light efficacy for inducing apoptosis in Candida albicans cultures. Roum Arch Microbiol Immunol.

[38] Behzadi P, Behzadi E (2011). A study on apoptosis inducing effects of UVB irradiation in Pseudomonas aeruginosa. Roum Arch Microbiol Immunol.

[39] Schaeffer EM (2017). Re: Escherichia coli Isolates from Patients with Bacteremic Urinary Tract Infection are Genetically Distinct from Those Derived from Sepsis following Prostate Transrectal Biopsy. J Urol.

[40] Yu M, Robinson K, Siegel C, Menias C (2017). Complicated Genitourinary Tract Infections and Mimics. Curr Probl Diagn Radiol.

[41] Leite JL, Rojas TCG, Maluta RP, de Silveira WD, Torres AG (10). Extra-Intestinal Escherichia coli (Uropathogenic E coli and Avian Pathogenic E coli). Escherichia coli in the Americas.

[42] Behzadi E, Behzadi P (2016). The role of toll-like receptors (TLRs) in urinary tract infections (UTIs). Cent European J Urol.

[43] Johnson JR, Russo TA (2018). Acute Pyelonephritis in Adults. N Engl J Med.

[44] Kaambo E, Africa C, Chambuso R, Passmore JS (2018). Vaginal Microbiomes Associated With Aerobic Vaginitis and Bacterial Vaginosis. Front Public Health.

[45] Loeper N, Graspeuntner S, Rupp J (2018). Microbiota changes impact on sexually transmitted infections and the development of pelvic inflammatory disease. Microbes Infect.

[46] Melman A (1983). The interaction of urinary tract infection and sexual intercourse in women. Sexual Disabil.

[47] Vahlensieck W, Perepanova T, Johansen TEB, Tenke P, Naber KG, Wagenlehner FM (2016). Management of uncomplicated recurrent urinary tract infections. Europ Urol Suppl.

[48] Tenney J, Hudson N, Alnifaidy H, Li JTC, Fung KH (2018). Risk factors for aquiring multidrug-resistant organisms in urinary tract infections: A systematic literature review. Saudi Pharm J.

[49] Rodríguez-Baño J, Gutiérrez-Gutiérrez B, Machuca I, Pascual A (2018). Treatment of Infections Caused by Extended-Spectrum-Beta-Lactamase-, AmpC-, and Carbapenemase-Producing Enterobacteriaceae. Clin Microbiol Rev.

[50] Spencer JD, Schwaderer AL, Becknell B, Watson J, Hains DS (2014). The innate immune response during urinary tract infection and pyelonephritis. Pediatr Nephrol.

[51] Gluba A, Banach M, Hannam S, Mikhailidis DP, Sakowicz A, Rysz J (2010). The role of Toll-like receptors in renal diseases. Nat Rev Nephrol.

[52] Reygaert WC (2014). Innate Immune response to urinary tract infections involving Escherichia coli. J Clin Cell Immunol.

[53] Behzadi P, Behzadi E (2006). Detection of Apoptosis Feature In Ultraviolet Light-Exposed Trichophyton rubrum. Turkiye Klinikleri J Med Sci.

[54] Kawai T, Akira S (2010). The role of pattern-recognition receptors in innate immunity: update on Toll-like receptors. Nat Immunol.

[55] Netea MG, Van Der Graaf CA, Vonk AG, Verschueren I, Van Der Meer JW, Kullberg BJ (2002). The role of toll-like receptor (TLR) 2 and TLR4 in the host defense against disseminated candidiasis. J Infect Dis.

[56] Philpott DJ, Girardin SE (2004). The role of Toll-like receptors and Nod proteins in bacterial infection. Mol Immunol.

[57] Chen K, Huang J, Gong W, Iribarren P, Dunlop NM, Wang JM (2007). Toll-like receptors in inflammation, infection and cancer. Int Immunopharmacol.

[58] Takeda K, Kaisho T, Akira S (2003). Toll-like receptors. Annu Rev Immunol.

[59] Sonnex C (2010). Toll-like receptors and genital tract infection. Int J STD AIDS.

[60] Dowling JK, Dellacasagrande J (2016). Toll-Like Receptors: Ligands, Cell-Based Models, and Readouts for Receptor Action. Methods Mol Biol.

[61] Uematsu S, Akira S (2006). Toll-like receptors and innate immunity. J Mol Med.

[62] Gordon NC, Wareham DW (2010). Multidrug-resistant Acinetobacter baumannii: mechanisms of virulence and resistance. Int J Antimicrob Agents.

[63] Gilchrist JJ, MacLennan CA, Hill AV (2015). Genetic susceptibility to invasive Salmonella disease. Nat Rev Immunol.

[64] Massari P, Toussi DN, Tifrea DF, de la Maza LM (2013). Toll-like receptor 2-dependent activity of native major outer membrane protein proteosomes of Chlamydia trachomatis. Infect Immun.

[65] Chang JH, Park JY, Kim SK (2006). Dependence on p38 MAPK signalling in the up-regulation of TLR2, TLR4 and TLR9 gene expression in Trichomonas vaginalis-treated HeLa cells. Immunology.

[66] Zariffard MR, Novak RM, Lurain N, Sha BE, Graham P, Spear GT (2005). Induction of tumor necrosis factor- alpha secretion and toll-like receptor 2 and 4 mRNA expression by genital mucosal fluids from women with bacterial vaginosis. J Infect Dis.

[67] Ma Y, He B (2014). Recognition of herpes simplex viruses: toll-like receptors and beyond. J Mol Biol.

[68] Armbruster CE, Smith SN, Mody L, Mobley HLT (2018). Urine cytokine and chemokine levels predict urinary tract infection severity independent of uropathogen, urine bacterial burden, host genetics, and host age. Infect Immun.

[69] Fritzenwanker M, Imirzalioglu C, Chakraborty T, Wagenlehner FM (2016). Modern diagnostic methods for urinary tract infections. Expert Rev Anti Infect Ther.

[70] Aragón IM, Herrera-Imbroda B, Queipo-Ortuño MI, Castillo E, Del Moral JS, Gómez-Millán J, Yucel G, Lara MF (2018). The Urinary Tract Microbiome in Health and Disease. Eur Urol Focus.

[71] Behzadi P, Ranjbar R (2018). DNA microarray technology and bioinformatic web services. Acta Microbiol Immunol Hung.

[72] Fouts DE, Pieper R, Szpakowski S, Pohl H, Knoblach S, Suh MJ, Huang ST, Ljungberg I, Sprague BM, Lucas SK, Torralba M, Nelson KE, Groah SL (2012). Integrated next-generation sequencing of 16S rDNA and metaproteomics differentiate the healthy urine microbiome from asymptomatic bacteriuria in neuropathic bladder associated with spinal cord injury. J Transl Med.

[73] Lewis DA, Brown R, Williams J, White P, Jacobson SK, Marchesi JR, Drake MJ (2013). The human urinary microbiome; bacterial DNA in voided urine of asymptomatic adults. Front Cell Infect Microbiol.

